# Continuous intra-gastral monitoring of intra-abdominal pressure in critically ill children: a validation study

**DOI:** 10.1186/s40635-021-00386-8

**Published:** 2021-05-24

**Authors:** T. Kaussen, M. Gutting, F. Lasch, D. Boethig, A. von Gise, J. Dingemann, H. Koeditz, T. Jack, M. Sasse, P. Beerbaum, M. Boehne

**Affiliations:** 1 Department of Paediatric Cardiology and Intensive Care Medicine, Hannover Medical School, Hannover, Germany; 2 Institute of Biometry, Hannover Medical School, Hannover, Germany; 3 Department of Paediatric Surgery, Hannover Medical School, Hannover, Germany; 4 Department of Paediatric Cardiology and Intensive Care Medicine, Hannover Medical School, University Children’s Hospital, Carl-Neuberg-Str. 1, 30625 Hannover, Germany

**Keywords:** Intra-abdominal pressure measurement, Intra-gastric pressure measurement, Air capsule nasogastric tube, Intra-abdominal hypertension, Abdominal compartment syndrome, Paediatric intensive care

## Abstract

**Background:**

In critically ill children, detection of intra-abdominal hypertension (IAH > 10 mmHg) and abdominal compartment syndrome (ACS = IAH + organ dysfunction) is paramount and usually monitored through intra-vesical pressures (IVP) as current standard. IVP, however, carries important disadvantages, being time-consuming, discontinuous, with infection risk through observer-dependent manipulation, and ill-defined for catheter sizes. Therefore, we sought to validate air-capsule-based measurement of intra-gastric pressure (ACM-IGP).

**Methods:**

We prospectively compared ACM-IGP with IVP both in vivo and in vitro (water column), according to Abdominal-Compartment-Society validation criteria. We controlled for patient age, admission diagnosis, gastric filling/propulsive medication, respiratory status, sedation levels and transurethral catheters, all influencing intra-abdominal pressure (IAP).

**Results:**

In tertiary care PICU setting, finally, *n* = 97 children were enrolled (median age, 1.3 years [range 0 days–17 years], LOS-PICU 8.0 [1–332] days, PRISM-III-Score 13 [0–35]). In *n* = 2.770 measurements pairs, median IAP was 6.7 [0.9–23.0] mmHg, *n* = 38 (39%) children suffered from IAH > 10 mmHg, *n* = 4 from ACS. In vitro against water column, ACM-IGP correlated perfectly (*r*^2^ 0.99, mean bias − 0.1 ± 0.5 mmHg, limits of agreement (LOA) − 1.1/+ 0.9, percentage error [PE] 12%) as compared with IVP (*r*^2^ 0.98, bias + 0.7 ± 0.6 mmHg, LOA − 0.5/+ 1.9, PE 15%). With larger IVP catheters at higher pressure levels, IVP underestimated pressures against water column. In vivo, agreement between either technique was strong (*r*^2^ 0.95, bias 0.3 ± 0.8 mmHg, LOA − 1.3/+ 1.9 mmHg, PE 23%). No impact of predefined control variables on measurement agreement was observed.

**Conclusions:**

In a large PICU population with high IAH prevalence, ACM-IGP agreed favourably with IVP. More widespread usage of ACM-IGP may improve detection rates of ACS in critically ill children.

*Trial registration* WHO-ICTRP-No. DRKS00006556 (German Clinical Trial Register). Registered 12th September 2014, URL: https://www.drks.de/drks_web/navigate.do?navigationId=trial.HTML&TRIAL_ID=DRKS00006556

**Supplementary Information:**

The online version contains supplementary material available at 10.1186/s40635-021-00386-8.

## Background

Abdominal compartment syndrome (ACS) in children is defined as sustained intra-abdominal hypertension (IAH, intra-abdominal pressure (IAP) > 10 mmHg) accompanied with organ dysfunction (new or deteriorating) [1]. Delay in recognition or treatment may increase the mortality of ACS up to 90% [2, 3]. ACS occurs in the context of abdominal disease, burns, trauma, sepsis or systemic inflammation. More specifically in paediatrics, congenital abdominal wall or diaphragm defects, organ transplantation and necrotizing enterocolitis may be predisposing disorders [4].

According to Abdominal Compartment Society (WSACS; formerly: World Society of Abdominal Compartment Syndrome) recommendations, measurement of intra-vesical pressure (IVP) is the current reference method of IAP determination in children [1]. In clinical practice, regular measurement of IAP remains the exception due to the fact that IVP recording is known to be time-consuming and observer-dependent as it requires manual handling, with associated work load, source of sampling error and risk of urinary tract infection [4]. Moreover, IVP measures discontinuously and therefore may not capture acute IAP changes. Experienced clinicians rely on their clinical “semi-quantitative” estimation of IAP through palpation. Unfortunately, this practice has been shown to only poorly correlate with quantitative IAP measurement [5, 6] and cannot replace it [7, 8].

**Fig. 1:**
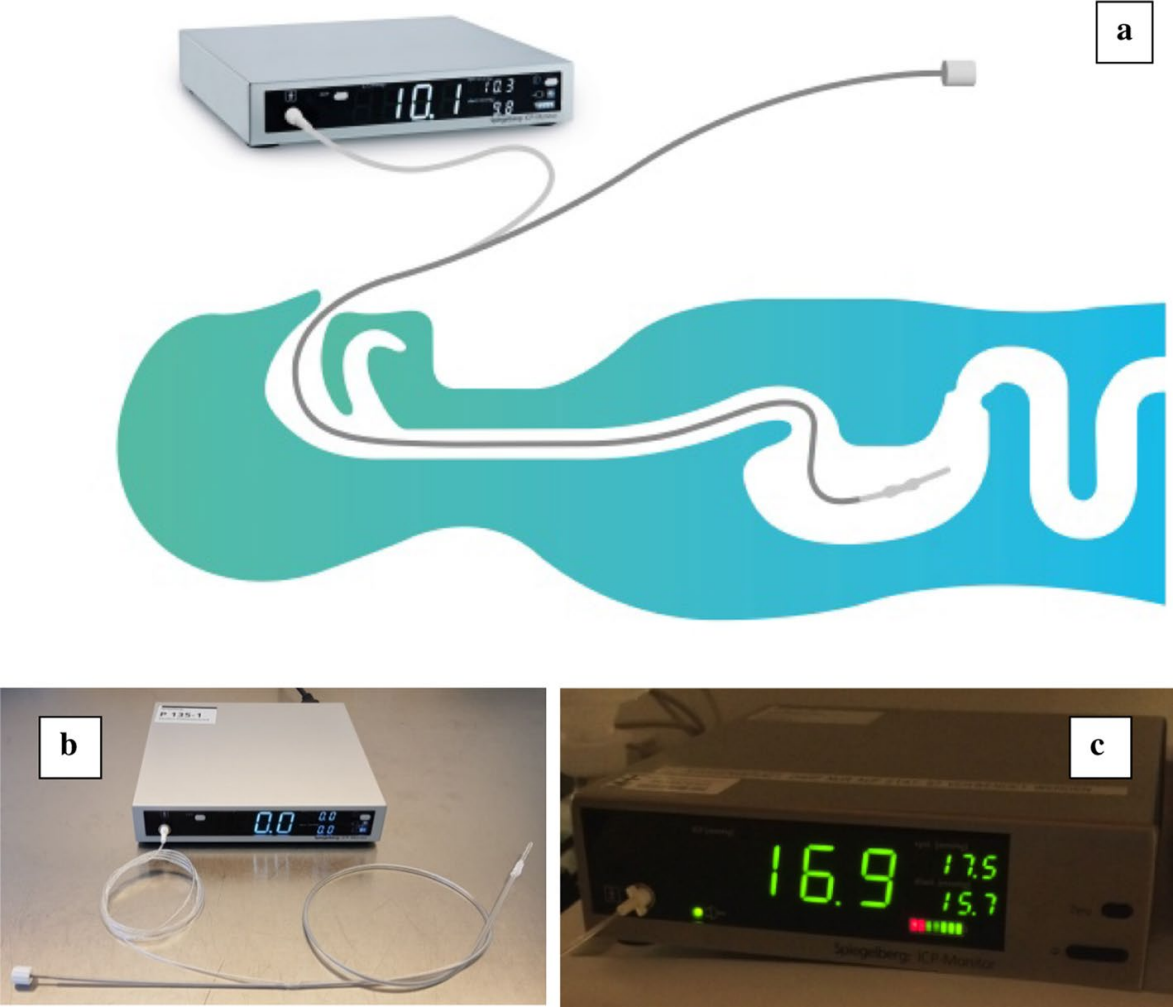
Illustration of air-capsule-based intra-abdominal pressure measurement system (ACM-IGP). **a** Schematic illustration of the customized catheter of the air-capsule-based measurement of intra-gastric pressure (ACM-IGP) system, which is equivalent to a special double-lumen 9F nasogastric tube, inserted into the stomach and connected to an ACM-IGP monitor (Illustration courtesy of Spiegelberg Company, Hamburg, Germany). The IAP normally undulates breath-synchronously (here: minimum 9.8 mmHg in expiration, maximum 10.3 mmHg in inspiration and 10.1 mmHg on average). Respiratory variations are considered as a quality criterion for IAP measurement; their absence indicates a malposition of the ACM-IGP or bladder catheter and usually requires their reinsertion. The insertion of an ACM-IGP catheter does not differ from that of a conventional nasogastric tube and is theoretically associated with a similar risk profile (malposition with aspiration, pneumonia, pneumothorax and esophageal or gastric perforation) [23, 24]. In patients beyond infancy, the placement is facilitated by an intraluminal guidewire provided by the manufacturer. In neonates and infants, the ACM-IGP catheter was placed without a guidewire, as the narrow bendig of the rigid guidewire in the pharynx hampers a later removal in this age group. All currently available ACM-IGP catheters do not have a radiopaque contrast. Therefore, the gastric catheter location was additionally verified by abdominal sonography in the present study. **b** Figure illustrates the ACM-IGP catheter connected to the ACM-IGP monitor. On the right side, the white, thin-skinned air capsule (sized 10 × 3 × 2.3 mm) is displayed at the gastric end of the ACM-IGP catheter, which is used for IAP measurement. The opposite side is connected to the pressure transducer on the left front of the ACM-IGP monitor. In the left lower margin the guide wire for insertion of the ACM-IGP catheter is displayed on the aboral end of the second lumen. Calibration and "zeroing" of the ACM-IGP system are fully automatic and repeated once per hour in the operating mode. During the continuous IAP measurement, the air capsule is filled with a defined air volume of 0.05–0.10 ml. Any pressure applied to the air capsule from outside is registered by the pressure transducer in the monitor and displayed as IAP with a precision of one decimal. **c** Illustration of a representative ACM-IGP measurement in a critically ill child with intra-abdominal hypertension (IAH) grade III (IAP = 16.9 mmHg). Please note that the displayed pressures with the minimum in exspiration (15.7 mmHg) and maximum in inspiration (17.5 mmHg) represent respiratory variations of IAP

Recently, an air-capsule-based measurement of intra-gastric pressure (ACM-IGP) has become available for continuous, fully automated, operator-independent IAP monitoring via a customized nasogastric tube (Fig. 1). This technique works through compression of an air-filled capsule with the pressure transmitted through an additional lumen of a nasogastric tube to an outside monitor. While increasingly used in the adult ICU medicine, the ACM-IGP technique has never been formally validated according to WSACS criteria, and certainly not in paediatric intensive care medicine (Additional file 1: Table S1) [9–11].

Therefore, we conducted a prospective, single-centre cohort study aiming to validate for the first time IAP measurements by ACM-IGP against IVP (reference method), utilizing all criteria for method validation as required by WSACS, both under initial steady-state conditions as well as longitudinally during the ICU stay. For both the initial and the longitudinal comparisons of ACM-IGP versus IVP, we explored the impact of prognostic variables of IAP measurement agreement, such as age, admission diagnosis, gastric filling status and gastrointestinal motility, respiratory status, sedation levels and IVP catheter. Moreover, we designed an in vitro experiment to investigate accuracy and precision of both methods using various catheter sizes against water column pressure recording representing a gold standard.

## Material and methods

### Study design

This prospective, longitudinal, observational single-centre study was conducted at the interdisciplinary PICU of Hannover Medical School (MHH) between January and August 2015. The clinical trial was approved by the local Ethics Committee (MHH-No. 6677) and registered internationally (WHO-ICTRP-No. DRKS00006556).

### Patient enrolment

All children admitted to PICU with a transurethral catheter and need for a nasogastric tube fulfilled the inclusion criteria and were enrolled provided that a steady-state IAP measurement condition could be achieved within the first 24 h after patient enrolment (see below). Exclusion criteria were premature birth, any diseases or malformations of the nasopharynx, upper gastrointestinal or urinary tract. Before enrolment, written informed consent was obtained from legal representatives.

### Clinical data collection

For each patient, demographic data at admission, diagnosis and length of stay at PICU (LOS-PICU) were recorded. To evaluate potential influencing factors on IAP measurement agreement between IVP and ACM-IGP, additional clinical data (patient age, admission diagnosis, gastric filling/propulsive medication, respiratory status, analgosedation levels, transurethral catheter type and size) were collected (for details refer to Additional file 1). Paediatric Risk of Mortality III Score (PRISM-III) was calculated on the first day of enrolment [12].

### Intra-abdominal pressure measurement (IAP)

Intra-vesical pressure (IVP) and intra-gastric pressure (IGP) measurements are considered indirectly determined correlates of intra-abdominal pressure (IAP). According to WSACS, an IAH was classified as IAP > 10 mmHg in at least two consecutive IVP measurements, an abdominal compartment syndrome (ACS) as IAH accompanied with organ dysfunction (new or deteriorating) [1, 13]. IAH was classified into four grades according to child-adapted WSACS criteria (I°: IAP > 10–12 mmHg, II°: 13–15 mmHg, III°: 16–18 mmHg, IV°: > 18 mmHg) [4].

#### Measurement practice

Based upon the modified Kron technique, IVP measurements were performed using a transurethral catheter according to WSACS recommendation (emptying the bladder, filling with 1 ml/kg BW normal saline [min. 3 ml, max. 25 ml] under aseptic conditions, waiting for at least 2 min to allow equilibration) with the midaxillary level as zero reference (clinical standard; [1, 14]). IVP is transmitted from the end-open transurethral catheter through the continuous liquid column in the catheter lumen to an outside pressure transducer (Codan, Germany). Transurethral catheters for IVP measurement were sizewise adjusted for weight and age (Norta-Nelaton 6–16 Charriére (Ch.) diameter, BSNmedical Company, Germany). For anatomical reasons, gastric tubes were used alternatively in small neonates (Flocare pursoft tube, 5 Ch., Nutricia Medical Devices, Netherlands).

IGP was determined by air-capsule-based measurement (ACM-IGP, Spiegelberg company, Germany) using a commercially available 9 French double-lumen nasogastric tube catheter with one separate lumen for continuous IGP measurement (please refer to Fig. 1) [9–11, 15, 16]. A thin air capsule (sized 10 × 3 × 2.3 mm) at the tip of the catheter is connected to a pressure transducer of a bedside ACM-IGP monitor. For IAP measurement, the air capsule is filled with a defined air volume of 0.05–0.10 ml through the ACM-IGP catheter lumen. Any variation of external pressure is immediately transduced via the air capsule to the monitor and converted into an electrical signal. The exact underlying technical process for IAP quantification is proprietary and at the discretion of the manufacturer.

The ACM-IGP system has been approved and CE-certified as an independent medical device for many years; all necessary tests were performed and its biocompatibility and endurance were confirmed ("shelf test"). The shelf life of the polyurethane catheter is specified by the manufacturer for 30 days.

For anatomical reasons, ACM-IGP catheters can generally be used for patients with a body weight of 3 kg or above. All ACM-IGP catheters were inserted nasally or perorally like conventional nasogastric tubes.

Using sonography, the correct positions of ACM-IGP and transurethral catheters were checked at least daily and additionally whenever ACM-IGP or IVP measurements showed no respiratory undulations. Such respiratory variations are considered as a quality criterion for IAP measurement; their absence indicate a malposition of the ACM-IGP or bladder catheter and usually require their reinsertion (please refer to Fig. 1).

#### Primary endpoint: initial agreement of ACM-IGP with IVP under steady-state conditions

For the primary goal—validation of ACM-IGP vs. IVP—the first simultaneous ACM-IGP and IVP measurements, once a steady-state condition of at least 5 min was achieved, were used for primary endpoint. Steady-state was defined as stable vital signs and analgosedation level (i.e. no movement of the patient, no change in the patient’s level of consciousness, stable heart rate and arterial pressure). Typically, the steady state was reached within the first hour of admission. For each patient, the first episode reaching the steady-state criteria was independently identified by two blinded, experienced paediatric intensive care physicians. In case of disagreement, a consensus was achieved with a third senior paediatric intensive care physician and the episode was allocated without ambiguity. All investigators were blinded for ACM-IGP and IVP measurements results. If a patient did not fulfil the steady-state criteria within 24 h after enrolment, a valid measurement could not be obtained and the patient was excluded from the study.

#### Secondary endpoint of agreement and explorative analysis of confounders under real-life conditions during the ICU stay

Following these primary comparison, all patients underwent longitudinal IAP measurements that were recorded simultaneously once per hour during daytime, for both evaluation of agreement and for explorative analyses investigating the potential impact of prognostic variables. Data were collected until either discharge from PICU, removal of IVP or ACM-IGP catheter, whichever came first. For these repeated measurements, taken during the patient’s recovery under reduced sedation, measurements were included in the analysis as long as there was no agitation and/or mass movement during recording. Additionally, measurements taken during ward rounds, dressing changes, rehabilitative therapies and other examinations or interventions were excluded.

### In vitro measurements

Comparable container models have been described earlier [9, 11, 17]. For details regarding the in vitro experimental set-up, refer to Additional file 1: Fig S1.

### Data processing and statistical analysis

Clinical data were recorded in a digital patient data monitoring system (Copra^®^Systems, Berlin, Germany), transferred to an Excel^®^2016 database (Microsoft^®^ Corporation, Redmond, USA) and analysed with SPSS^®^ Statistics V22.0 (IBM^®^, Armonk, North Castle, USA).

For in vivo and in vitro measurements, data of different IAP measurements methods were compared by linear regression analysis. Shapiro–Wilk testing revealed a non-normal data distribution; therefore, correlation analysis was performed by Spearman’s coefficient of determination. WSACS recommendations were applied to assess interchangeability of IAP measurement methods according to Bland–Altman (mean bias, limits of agreement [LOA], precision (standard deviation [SD] of the Bias) and percentage error [PE, LOA/mean IAP of both methods]) [18, 19]. Furthermore, a mean absolute percentage error (MAPE ± SD) was calculated according to de Myttenaere et al. [20].

#### Primary endpoint analysis

For the initial clinical baseline validation, the first pair of simultaneous IVP and ACM-IGP measurements under steady-state conditions of each enrolled subject was taken. To compare IVP and ACM-IGP measurements, the mean ${\mu }_{\mathrm{diff}}$ of the pairwise difference ${\mathrm{diff}}_{i}={\mathrm{ACMIGP}}_{i}- {\mathrm{IVP}}_{i}$ between IVP and ACM-IGP measurements was calculated (where the subscript *i* represents patient *i*).

To assess the agreement between both independent investigators, the pairwise differences (see above) for each patient were compared. To compare investigators A and B, the patient-specific difference between investigators was calculated as ${\mathrm{diff}}_{i}^{\mathrm{A}}- {\mathrm{diff}}_{i}^{\mathrm{B}}$, where ${\mathrm{diff}}_{i}^{\mathrm{A}}$ represents the difference between between IVP and ACM-IGP in patient *i* based on the assessment of investigator A. Using this difference, the mean difference between investigators was calculated as mean $\mathrm{mean}({\mathrm{diff}}_{i}^{\mathrm{A}}-\mathrm{ dif}{\mathrm{f}}_{i}^{\mathrm{B}})$ and the mean squared error as $\mathrm{mean} (({\mathrm{diff}}_{i}^{\mathrm{A}}-{\mathrm{diff}}_{i}^{\mathrm{B}}$)^2^).

#### Secondary endpoint analyses

For the longitudinal analysis of agreement during patient recovery, the initial and all pairs of simultaneous measurements were used. For the latter, we assumed that all measurement pairs were independent, because patient conditions varied much during ICU stay, regarding vital signs, vascular pressures, respiratory status and sedation levels.

In further exploratory analyses, the impact of clinical factors potentially influencing ACM-IGP agreement were evaluated separately for both the first pair of measures and for all longitudinal pairs of simultaneous IVP and ACM-IGP measurements.

## Results

### In vivo analysis

#### Patient characteristics

A population of *n* = 106 children met the inclusion criteria. Of those, *n* = 9 children did not fulfil steady-state criteria and were excluded from further analysis. Finally, *n* = 97 children (39% female) with a median age (range) of 1.3 years (0 days−17.0 years) could be enrolled (Table 1). Median PRISM-III score at admission was 13 (0−35), the overall mortality rate 8% and median LOS-PICU 8 (1−332) days. Admission diagnoses reflected a broad range of both post-operative and non-surgical entities as outlined in Table 2. As many as *n* = 38 of the 97 children (39%) suffered from IAH (I°: *n* = 27 [28%], II°: *n* = 9 [9%], III°: *n* = 2 [2%]), and *n* = 4 children (4%) showed ACS. During their stay on PICU, *n* = 92 (95%) children were temporarily mechanically ventilated. In addition to analgosedation, *n* = 13 (13%) patients received permanent neuromuscular blocking agents. About 50% of the children showed reduced peristalsis and about 1/3 were affected by gastric residuals, gastroparesis or subileus. *N* = 14 children (14%) required a temporary open-abdomen treatment.

**Table 1:** Patient demographics

Parameter	Number (%) or median (range)
Enrolled patients	97 (100%)
Female	38 (39%)
Age (years)	1.3 (0.0–17.0)
***Age category***	
Neonates	12 (12%)
Infants	29 (29%)
Toddlers	26 (27%)
School children	16 (17%)
Adolescents	14 (14%)
BMI (kg/m^2^)	15.6 (8.9–33.0)
Length of stay at PICU (days)	8 (1–332)
***Admission diagnoses***	
Cardiac and post-cardiac surgery	46 (47%)
Paediatric surgical	26 (27%)
Neurosurgical	9 (9%)
Traumatological	8 (8%)
***Non-surgical (internal)***	
Pulmonology	3 (3%)
Oncologic	3 (3%)
Infectious (sepsis)	2 (2%)
PRISM-III-score (first day of enrolment)	13.0 (0.0 – 35.0)
Prevalence of IAH overall	38 (39%)
IAH grade I (10–12 mmHg)	27 (28%)
IAH grade II (13–15 mmHg)	9 (9%)
IAH grade III (≥ 16 mmHg)	2 (2%)
Prevalence of ACS	4 (4%)
Mortality	8 (8%)

*BMI* body mass index, *PICU* paediatric intensive care unit, *PRISM-III-score* paediatric risk of mortality score III [12], *IAH* intra-abdominal hypertension, *ACS* abdominal compartment syndrome

**Table 2:** Results of primary and secondary analyses for in vivo measurement agreement of IVP and ACM-IGP methods in 97 paediatric patients

	Paired measurements per patient [median (range)]	IAP [median (range)]			Spearman correlation coefficient (*r*^2^)	WSACS method validation criteria					MAPE (SD) (%)
		ACM-IGP (mmHg)		IVP (mmHg)		No. of patients/measure pairs	Bias (mmHg)	Precision (mmHg)	LOA (mmHg)	PE (%)	
Target values^a^	–	–		–	≥ 0.6^b^	**> 20 subjects**	**≤ │1│**	**≤ 2**	***− 4 to + 4***	**≤ 25**	^c^
Primary analysis	1	6.8 (1.8–20.3)		6.0 (2.0–19.0)	0.95	97/97	0.3	0.8	***− ***1.3 to + 1.9	23	10 (11)
Secondary analysis	21 (1–132)	6.8 (0.9–23.0)	6.0 (1.0–20.0)		0.82	97/2770	0.3	1.2	− 2.1 to + 2.7	34	14 (16)

*ACM-IGP* air-capsule-based measurement of intra-gastric pressure, *Ch.* Charriére, *IAP* intra-abdominal pressure, *IVP* intra-vesical pressure, *LOA* limits of agreement, *MAPE* mean absolute percentage error, *No* number, *PE* percentage error, *SD* standard deviation, *WSACS* Abdominal Compartment Society (formerly: World Society of abdominal compartment syndrome)

^a^Target value specifications according to WSACS method validation criteria (bias + precision + LOA + PE) for the interchangeability of two IAP measurement methods [1]

^b^Spearman’s correlation coefficient (*r*^2^; target: *r*^2^ ≥ 0.6)

^c^Mean absolute percentage error (%) [19] were calculated in addition to recommended WSACS criteria [1]

Neither infections nor perforations of the upper digestive or urogenital tract were diagnosed as adverse events during IAP measurements.

#### IAP measurements

##### Primary analysis of first paired measurement (IVP versus ACM-IGP)

The first episode patients fulfilling the steady-state criteria was independently identified equally in 62 of 97 cases (64%) by the investigators; in 35 cases (36%) a consensus was achieved with a third investigator. The mean difference between the paired simultaneous IVP and ACM-IGP measurements selected by each investigator was − 0.03 ± 1.0 mmHg and the mean squared error was 1.0 ± 3.9mmHg^2^. Steady-state criteria were achieved in median after 2 (range 1−14) hours following enrolment.

**Fig. 2:**
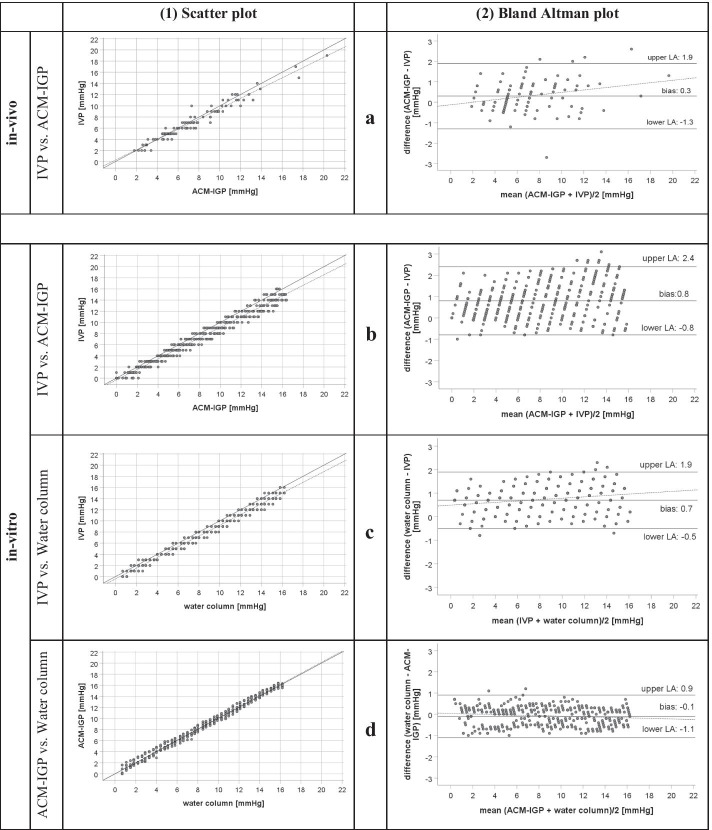
In vivo and in vitro measurements. Presentation of measurement agreement between IVP and ACM-IGP measurements in vivo (**a**) and in vitro (**b**–**d**). **a** Scatter plot and Bland–Altman plot of in vivo measurements. **a1** Scatter plot of paired IAP measurements obtained by novel ACM-IGP and IVP (reference method) with the solid line representing linear regression and the dashed line representing the line of identity. **a2** Bland–Altman plot of IVP and ACM-IGP. Mean bias ± precision between IVP and ACM-IGP was 0.3 ± 0.8 mmHg; limits of agreement (LOA) were − 1.3 to 1.9 mmHg. The dashed line represents the best-fit straight line, which increases slightly with rising IAP. **b**–**d** Scatter plots and Bland–Altman plots of in vitro measurements in a container model. In all scatter plots the solid line is representing linear regression and the dashed line the line of identity. **b1** Scatter plot of paired pressure measurements obtained by IVP and ACM-IGP. **b2** Bland–Altman plot: Mean bias ± precision was 0.8 ± 0.8 mmHg; LOA were − 0.8 to 2.4 mmHg. The dashed line represents the best-fit straight line, which is parallel to mean bias. **c1** Scatter plot of paired pressure measurements obtained by IVP and water column (gold standard). **c2** Bland–Altman plot: Mean bias ± precision was 0.7 ± 0.6 mmHg; LOA were − 0.5 to 1.9 mmHg. The dashed best-fit straight line increases slightly with rising pressures. **d1** Scatter plot of paired pressure measurements obtained by *ACM-IGP and water* column. **d2** Bland–Altman plot: Mean bias ± precision was − 0.1 ± 0.5 mmHg; LOA were − 1.1 to 0.9 mmHg. The dashed best-fit straight line increases slightly with rising pressures

The first *n* = 97 measurement pairs during steady state were included in the primary analysis. Median IAP (range) by IVP was 6.0 (2.0−19.0) mmHg and 6.8 (1.8−20.3) mmHg by ACM-IGP. A strong correlation (*r*^2^ = 0.95) was observed between both methods. Bland–Altman analysis between IVP and ACM-IGP revealed a mean IAP (± SD) of 7.1 ± 3.4 mmHg for both methods, a mean bias ± precision of 0.3 ± 0.8 mmHg with 95% limits of agreement (LOA) of − 1.3 and 1.9 mmHg (Fig. 2a, Table 2). Percentage error (PE) was 23% and mean absolute percentage error (MAPE) was 10 ± 11%.

##### Secondary analysis of longitudinal data (IVP versus ACM-IGP)

Totally, *n* = 4851 simultaneous IVP and ACM-IGP measurements were longitudinally performed in *n* = 97 subjects. *N* = 2081 measurements were recorded under agitation, mass movements, etc., and excluded from further analysis.

Finally, *n* = 2770 longitudinal measurement pairs with in median 21 (range 1−132) measurement pairs per child were recorded over 8 days in median (1–332) and further evaluated (Table 2). Median IAP (range) by IVP was 6.0 (1.0−20.0) mmHg, and 6.8 (0.9−23.0) mmHg by ACM-IGP. Spearman’s correlation coefficient between both methods was *r*^2^ = 0.82. The Bland–Altman analysis revealed a mean IAP of 7.1 ± 2.6 mmHg for both methods, a mean bias ± precision of 0.3 ± 1.2 mmHg, with LOA of − 2.1 and 2.7 mmHg (Table 2). The PE was 34%, MAPE was 14 ± 16%.

##### Explorative analyses for prognostic factors

The exploratory analyses of both, the first and the longitudinal paired measurements did not reveal any clinically relevant confounding factors with regard to patient age, respiratory status, analgosedation level, gastrointestinal motility and admission diagnosis (Additional file 1: Tables S2, S3).

### In vitro measurements

In the container model, 86 single measurements were performed in each of the four different test series. Thus, in total 344 measurements were compared between ACM-IGP versus water columns, IVP versus water columns and ACM-IGP versus IVP (Table 3).

**Table 3:** Results of the in vitro examination of measurement agreement of IVP and ACM-IGP technique compared to a water column in a container model

		Paired measurements	IAP mean (mmHg)	Spearman correlation coefficient (*r*^2^)	WSACS method validation criteria				MAPE (SD) (%)
Experimental arrangement					Bias (mmHg)	Precision (mmHg)	LOA (mmHg)	PE (%)	
	Target values according to [1]^a^	–		≥ 0.6^b^	**≤ │1│**	**≤ 2**	− ***4 to + 4***	**≤ 25**	^c^
Setting 1: gastric tube (5 Ch.)	ACM-IGP vs. water column	86	8.4	0.99	0.1	0.2	− 0.3 to 0.5	5	6 (13)
	Gastric tube (5 Ch.) vs. water column	86	8.0	0.99	0.8	0.3	0.2 to 1.4	8	17 (21)
	ACM-IGP vs. gastric tube (5 Ch.)	86	8.0	0.99	0.7	0.4	− 0.1 to 1.5	10	13 (14)
Setting 2: gastric tube (8 Ch.)	ACM-IGP vs. water column	86	8.3	0.99	0.3	0.2	− 0.1 to 0.7	5	8 (16)
	Gastric tube (8 Ch.) vs. water column	86	8.4	0.99	0.1	0.4	− 0.7 to 0.9	10	9 (18)
	ACM-IGP vs. gastric tube (8 Ch.)	86	8.2	0.99	− 0.2	0.4	− 1.0 to 0.6	10	9 (20)
Setting 3: transurethral catheter (6 Ch.)	ACM-IGP vs. water column	86	8.5	0.99	− 0.1	0.5	− 1.1 to 1.1	12	9 (13)
	Transurethral catheter (6 Ch.) vs. water column	86	8.0	0.99	0.9	0.5	− 0.1 to 1.9	13	16 (0)
	ACM-IGP vs. transurethral catheter (6 Ch.)	86	8.0	0.99	0.9	0.5	− 0.1 to 1.9	13	15 (12)
Setting 4: transurethral catheter (8 Ch.)	ACM-IGP vs. water column	86	8.7	0.99	− 0.6	0.2	− 1.0 to − 0.2	5	14 (22)
	Transurethral catheter (8 Ch.) vs. water column	86	7.9	0.99	1.1	0.6	− 0.1 to 2.3	15	19 (20)
	ACM-IGP vs. transurethral catheter (8 Ch.)	86	8.2	0.99	1.7	0.6	0.5 to 2.9	15	27 (16)
Overall	ACM-IGP vs. water column	344	8.5	0.99	− 0.1	0.5	− 1.1 to 0.9	12	9 (17)
	IVP (transurethral catheter + gastric tube) vs. water column	344	8.1	0.98	0.7	0.6	− 0.5 to 1.9	15	15 (20)
	ACM-IGP vs. IVP (transurethral catheter + gastric tube)	344	8.1	0.97	0.8	0.8	− 0.8 to 2.4	20	16 (17)

*ACM-IGP* air-capsule-based measurement of intra-gastric pressure, *Ch.* Charriére, *IAP* intra-abdominal pressure, *IVP* intra-vesical pressure, *LOA* limits of agreement, *MAPE* mean absolute percentage error, *No* number, *PE* percentage error, *SD* standard deviation, *WSACS* Abdominal Compartment Society (formerly: World Society of Abdominal Compartment Syndrome)

^a^Target value specifications according to WSACS method validation criteria (bias + precision + LOA + PE) for the interchangeability of two IAP measurement methods [1]

^b^Spearman’s correlation coefficient (*r*^2^; target: *r*^2^ ≥ 0.6)

^c^Mean absolute percentage error (%; MAPE [19]) were calculated in addition to recommended WSACS criteria [1]

The overall agreement between the height of the water column and pressures recorded by ACM-IGP (*r*^2^ 0.99, mean bias ± precision − 0.1 ± 0.5 mmHg, LOA − 1.1 to 0.9 mmHg, PE 12%, MAPE 9 ± 17%) and IVP technique (*r*^2^ 0.98, mean bias ± precision + 0.7 ± 0.6 mmHg, LOA − 0.5 to 1.9 mmHg, PE 15%, MAPE 16 ± 17%) was excellent (Fig. 2b–d, Table 3). Pressures obtained by ACM-IGP and IVP agreed well (*r*^2^ 0.97, mean bias ± precision 0.8 ± 0.8 mmHg, LOA − 0.8 to 2.4 mmHg, PE 20%, MAPE 15 ± 20%).

Interestingly, with both, gastric tubes (5 and 8 Ch.) and transurethral catheters (6 and 8 Ch.), the differences between pressures recorded by IVP technique and the height of the water column tended to increase with rising pressures (Table 3, Additional file 1: Fig. S3).

## Discussion

### Study design and key messages

In this study, the main objective was to validate the air-capsule-based measurement of intra-gastric pressure (ACM-IGP) in a clinical real-life paediatric ICU setting. Therefore, we conducted a prospective cross-sectional validation analysis of intra-abdominal pressure (IAP) in *n* = 97 critically ill children. Notably, for the first time, the present validation study fulfilled all requested WSACS criteria for comparison of different IAP measurement methods. We were able to gather reliable data across a wide age range, extending from neonatal to adolescent age, with adequate representation of all age groups. The PRISM-III scores (median 13, maximal 35) reflect a wide range of disease severity in our population. Our validation cohort is particularly valuable due to the high prevalence of intra-abdominal hypertension (IAH) of 39%, including IAH grade III (16–18 mmHg) and IAP levels up to 23 mmHg, with abdominal compartment syndrome (ACS) observed in 4% of all cases (Table 1, Additional file 1: Table S2). In this demanding population, we showed that the novel measurement technique ACM-IGP, is accurate, reproducible and robust when compared to the current clinical reference, namely intra-vesical pressure recording (IVP).

In our longitudinal analysis performed over a period of median 8.0 days (range 1–332) yielding 2770 measurement pairs, we further investigated associated clinical factors such as age, respiration, vigilance, peristalsis, gastric residuals and motility, or admission diagnoses, that may impact on measurement agreement of IAP. We did not find a relevant impact for these potential confounders on measurement agreement of ACM-IGP.

Interestingly, our in vitro validation of gastric ACM-IGP and IVP showed that ACM-IGP had an even better precision and accuracy than IVP against a water-column-based gold standard across a wide range of pressures as IVP slightly underestimated pressures particularly at higher levels, while the ACM-IGP method remained accurate. Clinically, the agreement between ACM-IGP and IVP while good in general seemed to slightly worsen at higher IAP levels.

Based on these in vitro and in vivo observations, we hypothesize that ACM-IGP may possibly be even better suited for IAP measurement, particularly in a clinical setting of high-risk for IAH.

### Motivation for the present study from previous work on IAP measurement

In 1981, Wesley et al. were the first to conceptualize the use of intra-gastric pressure (IGP) for IAP measurement [21]. The authors applied a water manometer through a gastrostomy tube during congenital abdominal wall repair in premature and neonates [21]. Another technique, a water-filled nasogastric tube connected to a pressure transducer was used by Davis et al. in 2005 for IGP measurements in children [22]. IGP correlated well with IAP directly assessed via a peritoneal dialysis catheter over a physiological pressure range of 1–8 mmHg [22]. Schachtrupp et al. first applied the air-capsule-based measurement (ACM) catheter in an animal validation study [15]. Instead of intra-gastric application—as provided for measurements in our study—they used an intra-abdominal location [15]. IAP derived by ACM method showed a stronger correlation compared to the laparoscopic insufflator gold standard than simultaneous IVP measurements—even at extremely high IAP levels [15]. Otto and colleagues transferred this experimental approach of intra-abdominally placed ACM to adult ICU patients [10]. ACM-derived IAP agreed well with IVP technique in elevated IAP up to 17 mmHg [10]. While these four landmark studies either applied IGP measurements via self-made devices, or used ACM placed intra-abdominally instead of intra-gastrically, Wauters et al. were the first who combined ACM with IGP determination in an animal model. Validated against an intra-abdominally placed fluid-filled catheter, ACM-IGP revealed excellent accuracy [16]. These encouraging data all motivated us to move from animal to clinical setting to conduct the first validation study using ACM with intra-gastric placement in a large setting of critically ill children presenting high IAH prevalence.

### Clinical implications

Both measurement methods reflect the IAP very accurately and are well tolerated in clinical practice. Handling of an ACM-IGP catheter is similar to that of a conventional nasogastric tube and therefore theoretically bears a comparable risk profile (malposition with aspiration, pneumonia, pneumothorax and esophageal or gastric perforation) [23, 24]. Nevertheless, to date, none of such complications have been published for the ACM-IGP system, nor have they been reported to the manufacturer nor were experienced in our institution in the last several years during multiple usage.

From a practical point of view ACM-IGP has the advantage that no infection-endangering bladder filling is necessary for IAP measurement [25, 26]. In addition, patient safety is enhanced by the fact that (1) the measurement is continuous; (2) medical staff is relieved; (3) the measurement method is more widely accepted due to its clinical-practical advantages, and (4) as a result of the more regular monitoring, IAP increases can be detected at an earlier stage and treated adequately in time. An earlier diagnosis of IAH in combination with a standardized therapeutic regime has recently shown to reduce the incidence of ACS from 10 to 2% in critically ill adults. The ACM-IGP method could facilitate the development and widespread implementation of a standardized diagnostic–therapeutic algorithm to reduce the incidence and morbidity of IAH and ACS in critically ill patients.

### Limitations of the study

For secondary and exploratory analyses, we pooled all longitudinal paired measurements, disregarding the fact that some were taken from identical individuals. We consider this as plausible since patient conditions varied much during ICU stay, regarding vital signs, vascular pressures, respiratory status, sedation levels and many other factors. The variations introduced by these different condition combinations outweigh the fact that they derive partly from the same patients. The results of the primary, secondary and exploratory analyses showed no relevant differences. Both addressed and non-addressed influencing factors were therefore not able to impair the strong measurement agreement between both methods.

## Conclusion

Our data allow the conclusion that both methods, IVP and ACM-IGP, reflect the IAP equally well. From clinical-practical and theoretical considerations, ACM-IGP may have advantages over the IVP technique.

With the help of this continuous monitoring method, timely diagnosis could be made easier and an adequate therapy could be initiated earlier in the future. Further prospective multicenter studies will be necessary to confirm the potential benefits of ACM-IGP in IAH diagnosis and therapy.

## Supplementary Information

**Additional file 1: **Continuous Intra-Gastral Monitoring of Intra-Abdominal Pressure in Critically ill Children – A Validation Study.

## Data Availability

Raw data and research results can be obtained from the authors upon reasonable request.

## Abbreviations

ACM-IGP: Air capsule nasogastric tube
ACS: Abdominal compartment syndrome
Ch.: Charriére
CPAP: Continuous positive airway pressure
IAH: Intra-abdominal hypertension
IAP: Intra-abdominal pressure
IGP: Intra-gastric pressure
IVP: Intra-vesical pressure
LOS-PICU: Length of stay at paediatric intensive care unit
MHH: Hannover Medical School
PICU: Paediatric intensive care unit
PRISM-III-Score: Paediatric Risk of Mortality III Score
WSACS: World Society of Abdominal Compartment Syndrome (recently re-named as: The Abdominal Compartment Society)
