# Supplementary material for: Continuous intra-gastral monitoring of intra-abdominal pressure in critically ill children: a validation study
Source: Intensive Care Med Exp. 2021 May 24;9:24. doi: 10.1186/s40635-021-00386-8 (PMC8141480; doi:10.1186/s40635-021-00386-8)
Supplement: Supplementary file 1 — Additional file 1: Continuous Intra-Gastral Monitoring of Intra-Abdominal Pressure in Critically ill Children – A Validation Study. [file 40635_2021_386_MOESM1_ESM.docx]

Additional File

# Introduction

### Tab. S1 Overview of validation studies on intra-abdominal pressure (IAP) measurement via an intra-gastric approach with focus on air-capsule-based measurement (ACM) systems


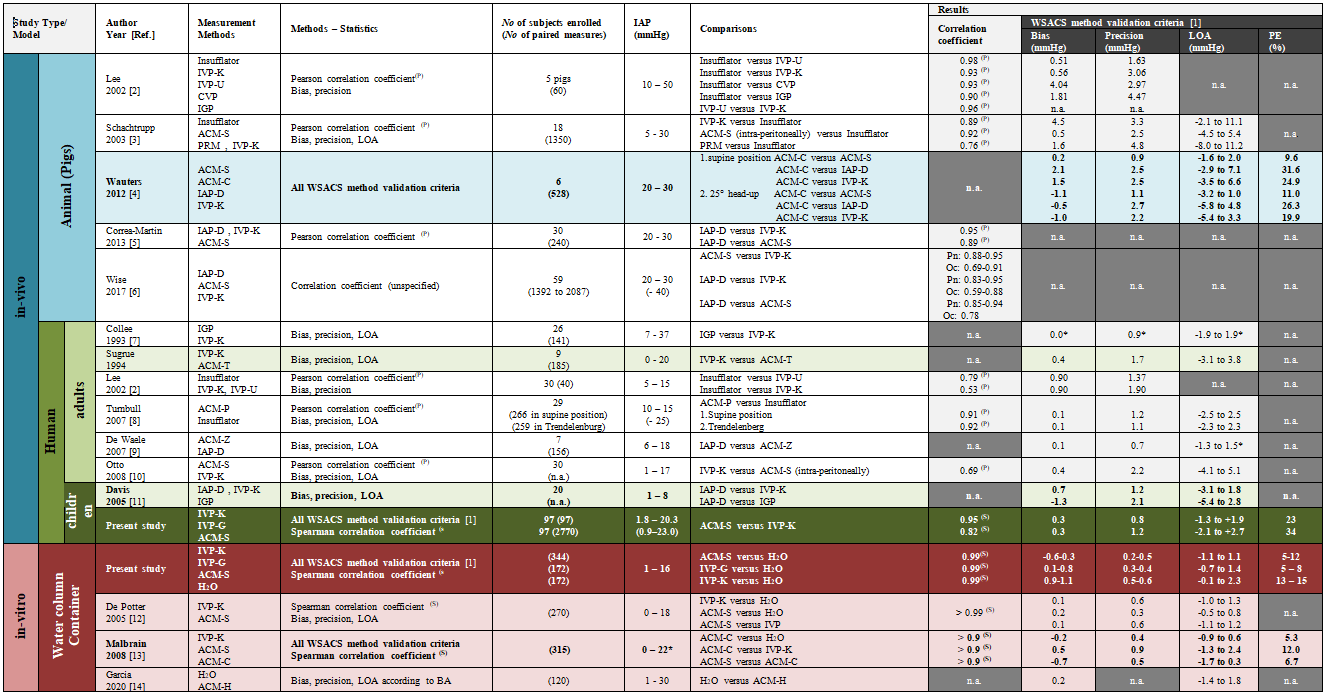


***Abbrev.:*** ACM: air-capsule-based measurement; BA: Bland-Altman [16]; Ch.: Charriére; CVP: central venous pressure; H_2_O: water column; IAH: Intra-abdominal hypertension; IAP: intra-abdominal pressure; IGP: intra-gastric pressure; IVP: intra-vesical pressure; LOA: limits of agreement; MAPE: mean absolute percentage error; No: number; Oc: Intestinal occlusion; PE: percentage error; Pn: Pneumoperitoneum; PRM: Piezoresistive pressure measurement; SD: standard deviation; WSACS: Abdominal Compartment Society (formerly: World Society of Abdominal Compartment Syndrome)

***Acknowledgements:*** † Measuring technique is currently not commercially available

***IAP measurement devices/ methods via the stomach:***

IGP: Intra-gastric pressure measurement via simple nasogastric tube

ACM-IGP: IGP using Air-capsule measurement technique

- ACM-S: Spiegelberg Enterprises, Hamburg, Germany
- ACM-C: Maquet Enterprises, Pulsion, Feldkirchen, Germany†
- ACM-Z: International Medical Systems, Zutphen, Netherlands†
- ACM-P: Ackrad Laboratories, Cranford, NJ, USA†
- ACM-T: Tonometric Inc, Bethesda, Maryland, USA†
- ACM-H: Handcrafted catheter

***IAP measurement methods other than via the stomach:***

PRM: Kodiag device, Braun-Dexon Enterprises, Tuttlingen, Germany†

IVP-K: Kron´s technique of IVP measurement using transurethral catheter

IVP-G: Kron´s technique of IVP measurement using gastric tube

IVP-U: U-tube technique of intra-vesical pressure measurement

IAP-D: Direct intraperitoneal measurement

CVP: Inferior caval vein pressure measurement

***IAH models:***

H_2_O: Water column in the container to simulate IAH

Pn: Pneumoperitoneum model with different IAP´s

Oc: Intestinal obstruction model with different IAP´s

# Material and Methods

## Clinical data collection

For each patient, demographic data at admission, diagnosis and length of stay at PICU (LOS-PICU) were recorded. To evaluate potential influencing factors on IAP measurement agreement between IVP and ACM-IGP, additional clinical data (patient age, admission diagnosis, gastric filling/propulsive medication, respiratory status, analgosedation levels, transurethral catheter type and size) were collected: Patients were classified into five age groups, namely neonates [0 days to 1 month], infants [1 month to 1 year], toddlers [2 to 5 years], schoolchildren [6 to 12 years] and adolescents [13 to <18th years]. Respiratory support was classified into 4 levels: spontaneous breathing, air or oxygen via nasal cannula, continuous positive airway pressure (CPAP), and mechanical ventilation (biphasic positive airway pressure). Analgosedation was assigned to 3 categories (no analgosedation - analgosedation - analgosedation plus neuromuscular blockade). Gastrointestinal motility was defined as abnormal when either drugs stimulating peristalsis were prescribed or gastric residuals were present. A 4-step graduation was performed depending on the number of administered drugs (0-3). The amount of gastric residuals was related to body weight (BW) and divided into 4 groups (no residuals; >0-5ml/kg BW; >5-10ml/kg BW; >10ml/kg BW).

## Intra-abdominal pressure measurement (IAP)

## In-vitro measurements

Modified heart catheter sheaths {3} (curved brackets {} refer to the numbers in ESM-Fig. 1) were inserted at the base of 25cm high and 15cm diameter tubular plastic containers {1}, through which the various ACM-IGP {4} or IVP measuring catheters and tubes {5} were inserted (ESM Fig. 1).

The air capsule of the IAP catheter {4} of the ACM-IGP system was inserted into the container via the sheath {3}, and the catheter connected to the associated ACM-IGP monitor {2} (automatic calibration and zero point definition takes place after switching on the monitor).

To test the IVP method, the tip comprising the balloon portion of the transurethral catheter or gastric tube {5} was placed into the container via the sheath {3}. The opposite side was connected via an adapter {6} to a pressure transducer {7], which in turn was connected to a patient monitor {not shown}. The transurethral catheter, the gastric tube {5} and the pressure transducer system {7} were previously filled completely with physiological saline solution without bubbles, so that pressure could be transferred from the container water column {1} to the pressure transducer {7} via a continuous fluid column. The patient monitor was zeroed before starting the actual pressure measurement. To simulate increasing IAPs of different heights, the container was slowly filled with normal saline in 0.5 cm water column steps (measured using a centimeter tape measure {8} attached to the outside wall of the container {1}). The resulting water column defined the pressure exerted on the various transurethral catheters and gastric tubes. The resulting water column (measured in cm {8} and converted into mmHg) was compared and related to the pressures determined and displayed by the ACM-IGP system (Spiegelberg® monitor {2}) on the one hand and by the patient monitor of the IVP measuring system on the other hand. Comparative measurements were carried out in 0.5 cm steps between 1 and 22 cmH_2_O (0.7 - 16.2 mmHg)


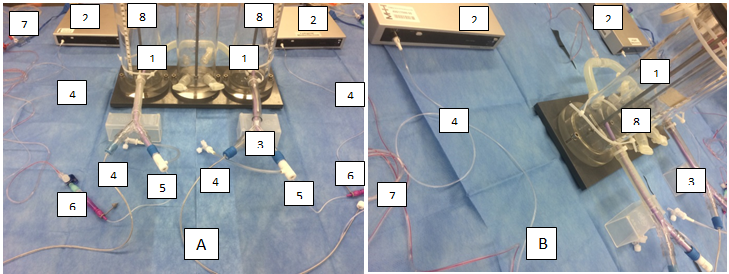


**Fig. S1 Experimental set-up of the in-vitro container model used to compare the measurement agreement of IVP and ACM-IGP methods with the pressure of a water column (gold standard)**

Fig. (A) and (B): Photographs of the experimental set-up from different perspectives with identification and description of the individual components (The curved brackets refer to the marked components shown in Fig. (A) and (B): {1} Plastic container – {2} ACM-IGP-monitor – {3} sheath – {4} IAP-catheter – {5} transurethral catheter/ gastric tube – {6} Adapter/ connector – {7} pressure transducer – {8} Centimeter measuring tape)

# Results

## In-vivo analysis

### Explorative analyses for confounding factors

Considering the WSACS validation criteria [1], the exploratory analyses of both, the first and the longitudinal paired measurements did not reveal any clinically relevant confounding factors with regard to patient age, respiratory status, analgosedation level, gastrointestinal motility and admission diagnosis (ESM-Tab. 2+3, see below).

Bland-Altman analysis of the exploratory primary analysis data of the first measurement pairs showed a bias between -0.1 to 0.6 mmHg with a precision of 0.3 to 1.4 mmHg. The lower limits of agreement (LOA) varied between -2.7 and 0.0 mmHg, the upper LOA between 1.2 to 2.9 mmHg with percentage errors between 12 and 32% or mean absolute percentage errors between 6 and 16%, respectively. The correlation coefficients r^2^ ranged between 0.84 and 0.98, thus showing a very strong correlation (ESM-Tab. 2, see below).

Bland-Altman analysis of the exploratory secondary analysis data of the longitudinal measurements revealed a bias ranging from 0.2 to 0.6 mmHg with a precision of 0.9 to 1.4 mmHg. Lower LOA varied between -2.3 and -1.4mmHg, the upper LOA between 2.2 to 3.4mmHg with percentage errors from 23 to 39% and mean absolute percentage errors between 11 and 16%. The correlation coefficients ranged between 0.74 and 0.91, thus showing a very strong correlation (ESM-Tab. 3, see below).

However, transurethral catheters size may have an influence on measurement agreement as the differences between IVP and ACM-IGP tended to increase with higher IAP and larger transurethral catheters (ESM-Tab. 2; ESM-Fig. 2, see below).

|  |  | **[1] Scatter plot** |  | **[2] Bland-Altman plot** |
| --- | --- | --- | --- | --- |
| **Gastric tube** | **5 Ch.** | **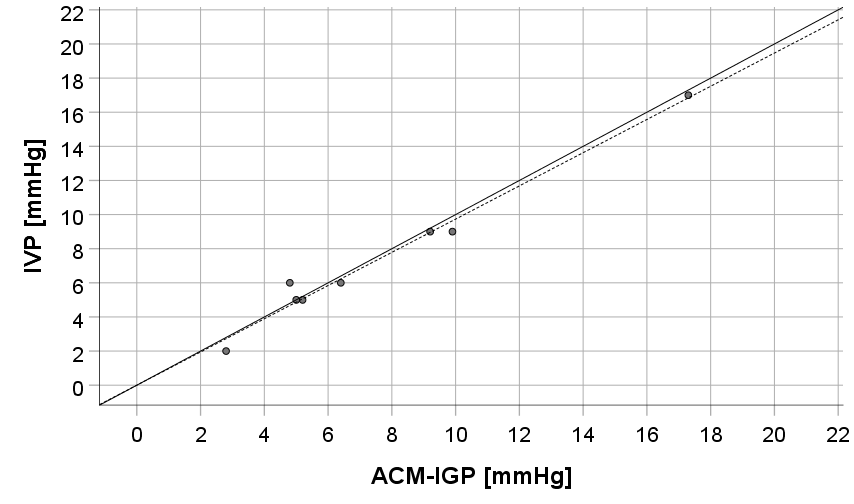** | **A** | **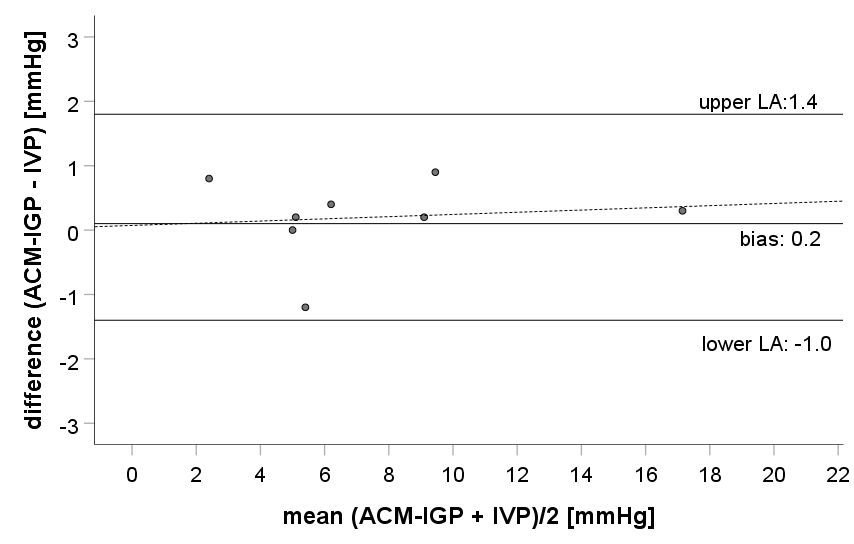** |
| **Transurethral catheters** | **6 Ch.** | **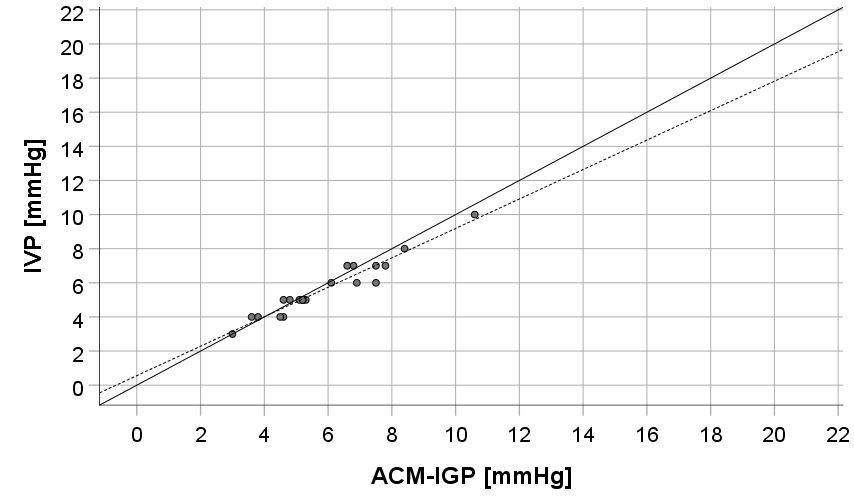** | **B** | **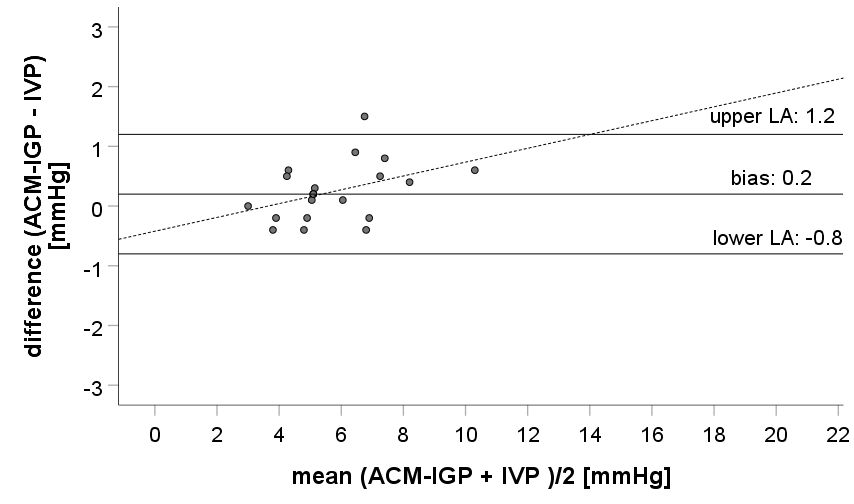** |
|  | **8 Ch.** | **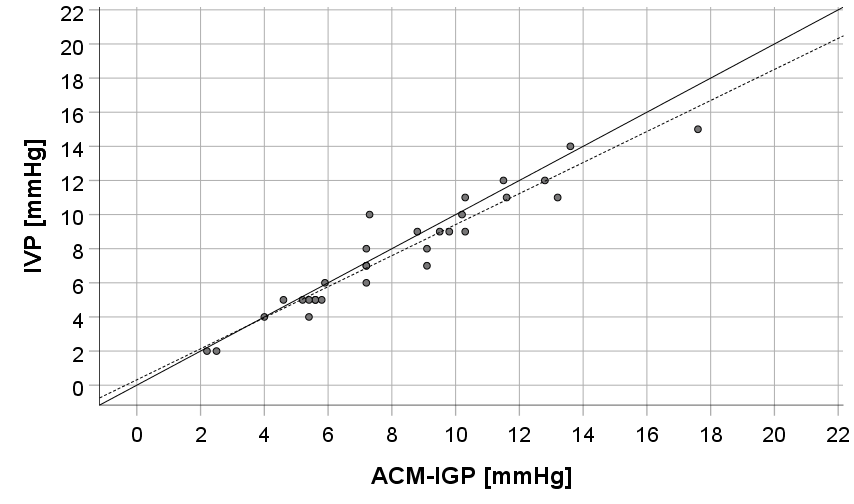** | **C** | **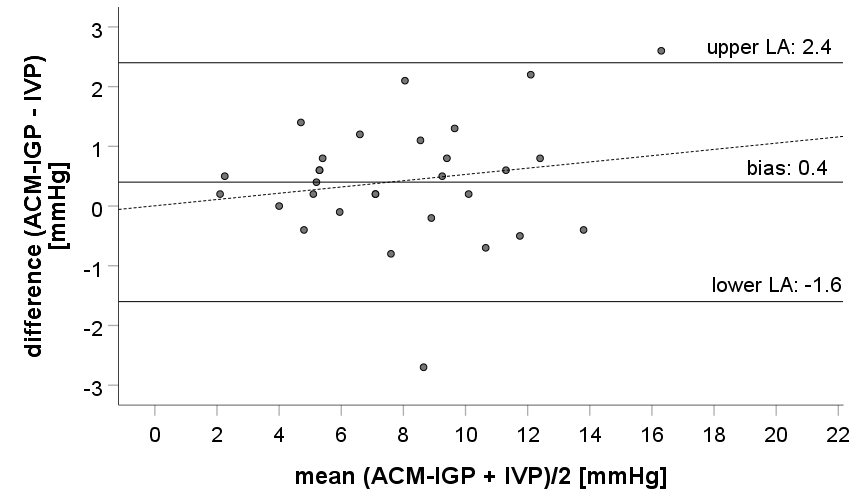** |
|  | **10 Ch.** | **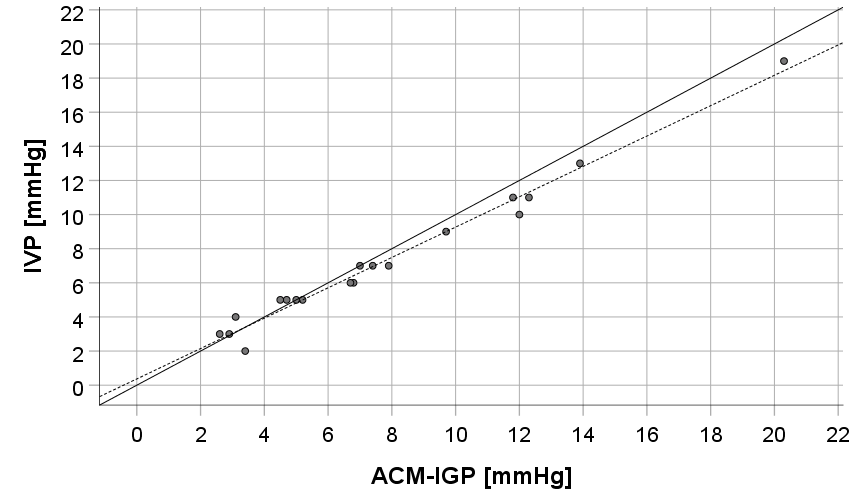** | **D** | **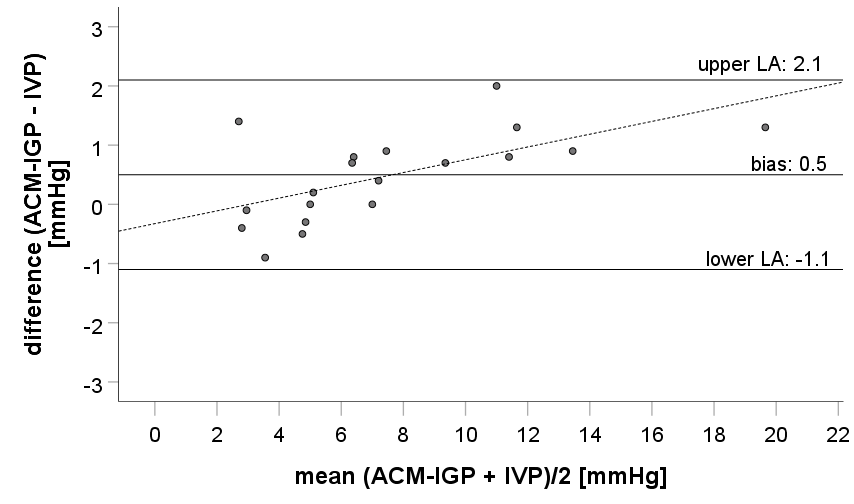** |
|  | **12 - 16 Ch.** | **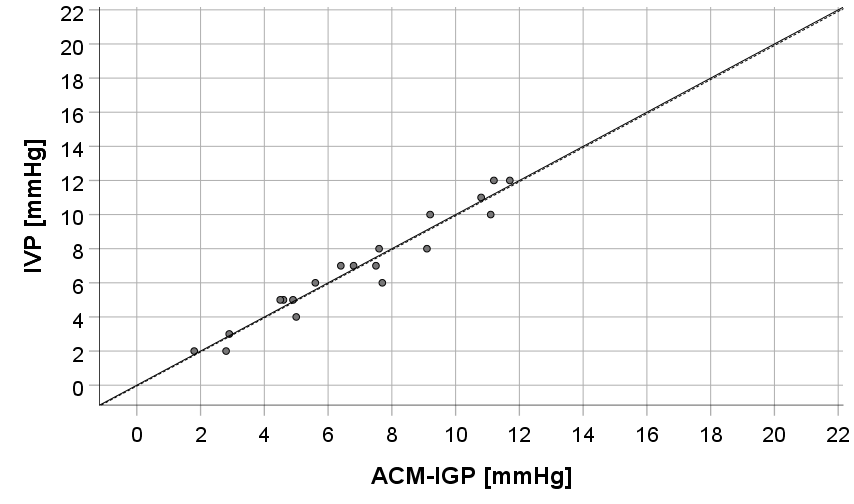** | **E** | **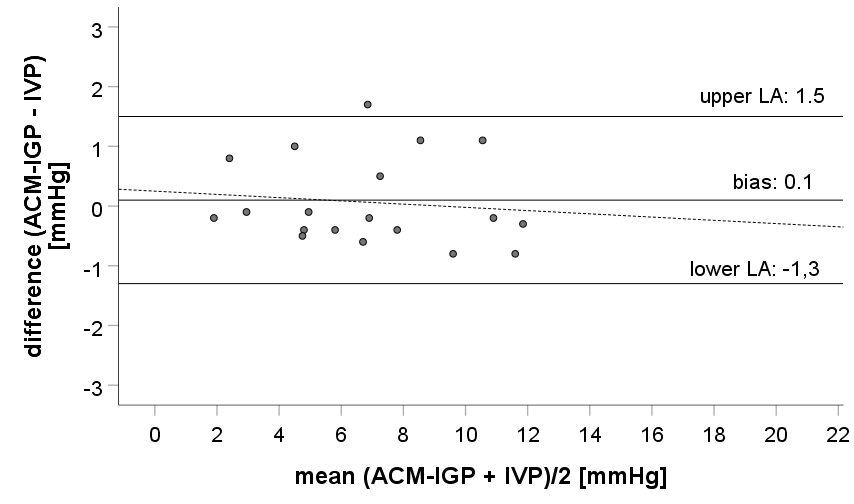** |

### Fig. S2: Scatter and Bland-Altman plots of paired *in-vivo* IAP measurements (IVP versus ACM-IGP) stratified by gastric tube or transurethral catheter of different sizes (based upon primary analysis data of the first paired measurements)

(A) Scatter plot and Bland-Altman plot of paired **in-vivo** ACM-IGP and IVP measurements via **gastric tube sized 5 Ch**. (A1) Scatter plot of paired IAP measurements (ACM-IGP vs. 5 Ch. gastric tube) with the solid line representing linear regression and the dashed line representing the line of identity. (A2) Bland–Altman plot of ACM-IGP and IVP (via 5 Ch. gastric tube). Mean Bias between IVP and ACM-IGP was 0.2 mmHg; precision (SD of the bias) was 0.6 mmHg; Limits of agreement (LOA) were -1.0 to 1.4 mmHg. Percentage error (PE) yielded for 16% and mean absolute percentage error (MAPE±SD) for 11±14%.

(B) Scatter plot and Bland-Altman plot of paired **in-vivo** ACM-IGP and IVP measurements via **transurethral catheter sized 6 Ch.** (B1) Scatter plot with the solid line representing linear regression and the dashed line representing the line of identity. (B2) Bland–Altman: Mean bias±precision 0.2±0.5mmHg; LOA -0.8 to 1.2 mmHg. PE 18%, MAPE±SD 7±6%.

(C) Scatter plot and Bland-Altman plot of paired **in-vivo** ACM-IGP and IVP measurements via **transurethral catheter sized 8 Ch**.. (C1) Scatter plot with the solid line representing linear regression and the dashed line representing the line of identity. (C2) Bland–Altman plot: Mean bias±precision 0.4±1.0 mmHg, LOA -1.6 to 2.4 mmHg, PE 25%, MAPE±SD 11±9 %.

(D) Scatter plot and Bland-Altman plot of paired **in-vivo** ACM-IGP and IVP measurements via **transurethral catheter sized 10 Ch**.. (D1) Scatter plot with the solid line representing linear regression and the dashed line representing the line of identity. (D2) Bland–Altman plot: Mean bias±precision 0.5±0.8 mmHg, LOA -1.1 to 2.1 mmHg, PE 21%, MAPE±SD 12±15 %.

(E) Scatter plot and Bland-Altman plot of paired **in-vivo** ACM-IGP and IVP measurements via **transurethral catheter sized 12 - 16 Ch.**. (E1) Scatter plot with the solid line representing linear regression and the dashed line representing the line of identity. (E2) Bland–Altman plot: Mean bias±precision 0.1±0.7 mmHg, LOA -1.3 to 1.5 mmHg, PE 20%, MAPE±SD 11±10%.

### Tab. S2: Exploratory Bland-Altman analysis of the first measurement pairs focussing on prognostic variables of IAP measurement agreement


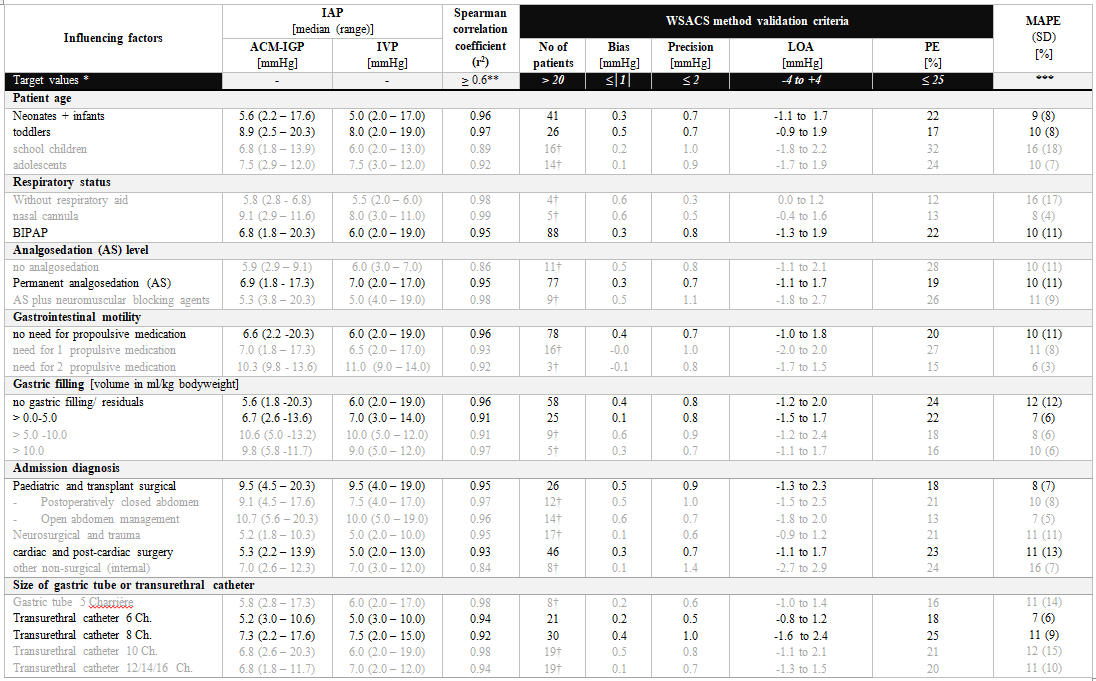


**Abbrev.:** ACM-IGP: air-capsule-based measurement of intra-gastric pressure; Ch.: Charriére; IAP: intra-abdominal pressure; IVP: intra-vesical pressure; LOA: limits of agreement; MAPE: mean absolute percentage error; No: number; PE: percentage error; SD: standard deviation; WSACS: Abdominal Compartment Society (formerly: World Society of Abdominal Compartment Syndrome)

**Acknowledgements:**

* Target value specifications according to WSACS method validation criteria (bias + precision + LOA + PE) for the interchangeability of two IAP measurement methods [1]

** Spearman´s correlation coefficient (r^2^; target: r²≥0.6) and *** Mean Absolute Percentage Error (%, MAPE [17]) were calculated in addition to recommended WSACS criteria [1].

**Tab. S3 Exploratory Bland-Altman analysis of the longitudinal data focussing on prognostic variables of IAP measurement agreement**


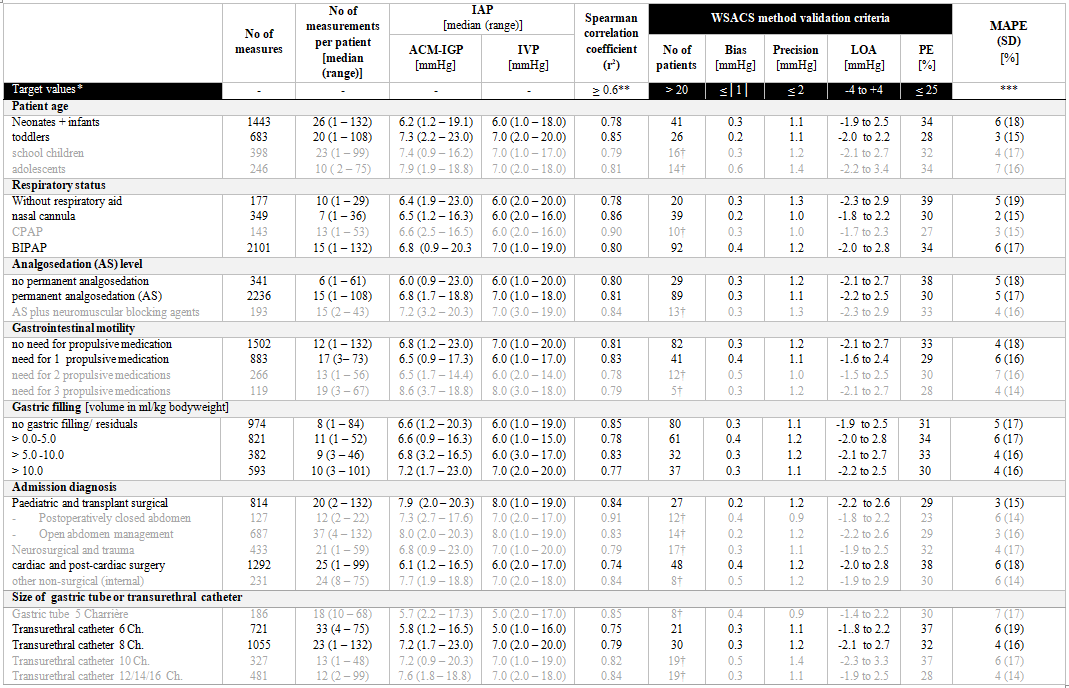


**Abbrev.:** ACM-IGP: air-capsule-based measurement of intra-gastric pressure; BIPAP: biphasic positive airway pressure (= pressure controlled mechanical ventilation); Ch.: Charriére; IAP: intra-abdominal pressure; CPAP: continuous positive airway pressure**;** IVP: intra-vesical pressure; LOA: limits of agreement; MAPE: mean absolute percentage error; No: number; PE: percentage error; SD: standard deviation; WSACS: Abdominal Compartment Society (formerly: World Society of Abdominal Compartment Syndrome)

**Acknowledgements:**

* Target value specifications according to WSACS method validation criteria (bias + precision + LOA + PE) for the interchangeability of two IAP measurement methods[1]

** Spearman´s correlation coefficient (r^2^; target: r²≥0.6) and *** Mean Absolute Percentage Error (%, MAPE [17]) were calculated in addition to recommended WSACS criteria[1] .

†Results of the respective subgroup, written in grey and marked with †, can only be considered as a tendency, since the min. number of 20 subjects recommended by the WSACS was not reached in this subgroup.

### Fig. S3: Graphical representation of the measurement agreement of *in-vitro* pressure measurements with IVP and ACM-IGP methods compared to the water column in a container model as a function of gastric tube or transurethral catheters of different sizes

|  |  | **[1] Scatter plot** |  | **[2] Bland-Altman plot** |
| --- | --- | --- | --- | --- |
| **Gastric tubes** | **5 Ch.** | **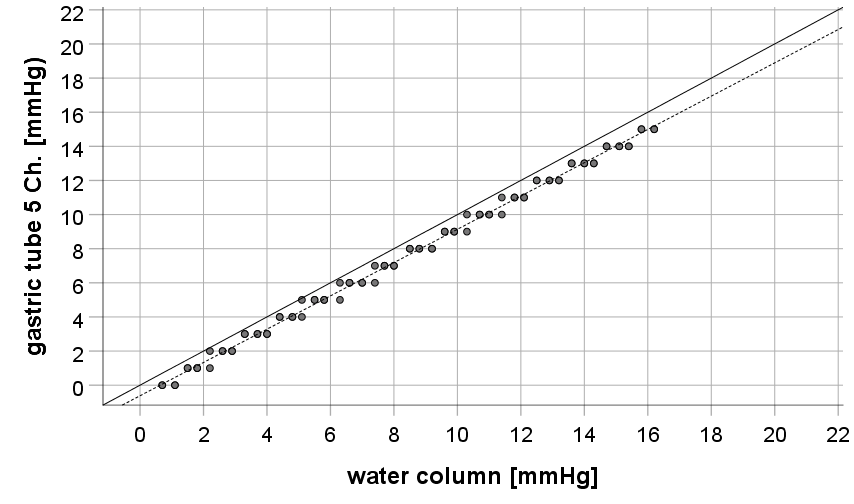** | **A** | **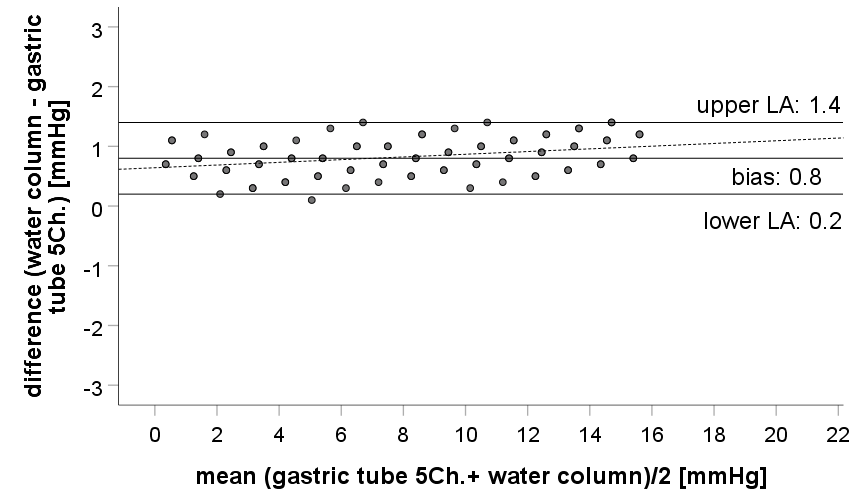** |
|  | **8 Ch.** | **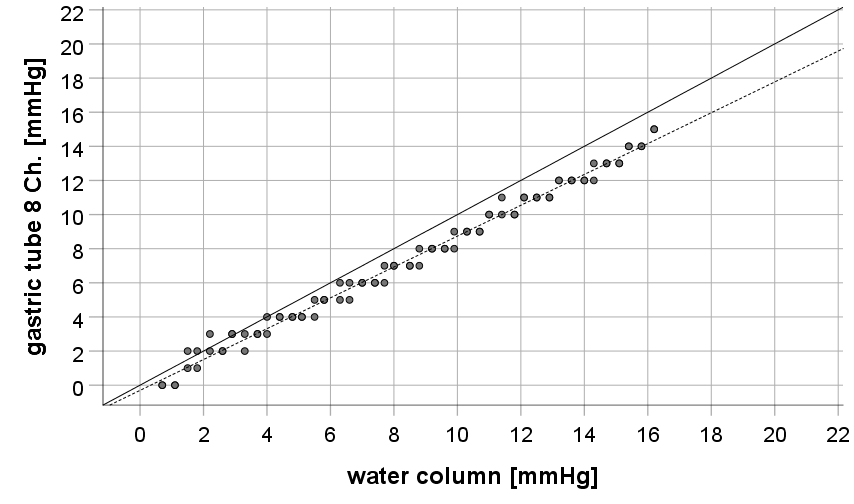** | **B** | **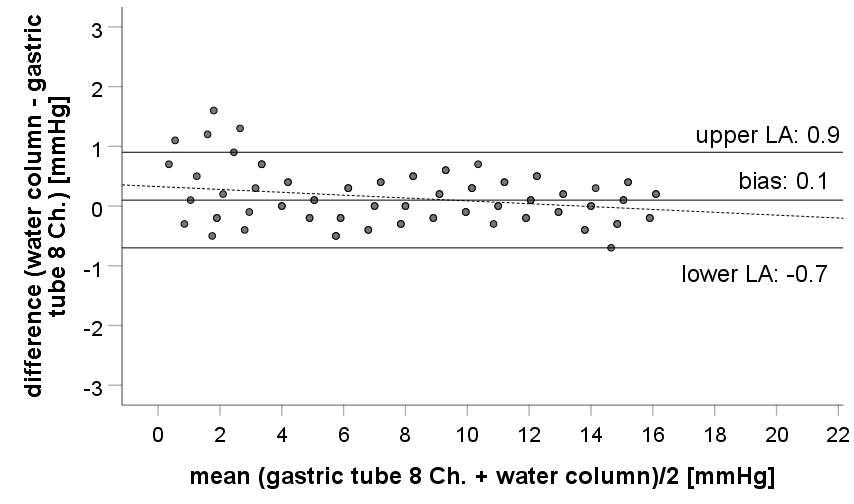** |
| **Transurethral catheters** | **6 Ch.** | **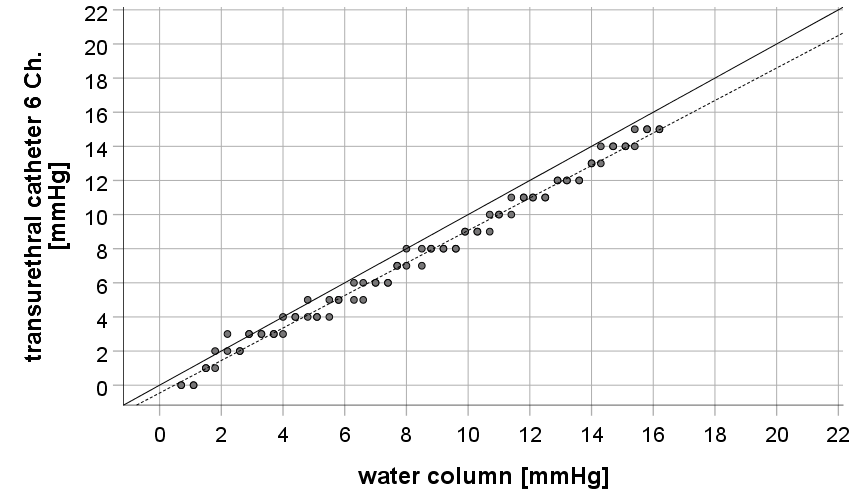** | **C** | **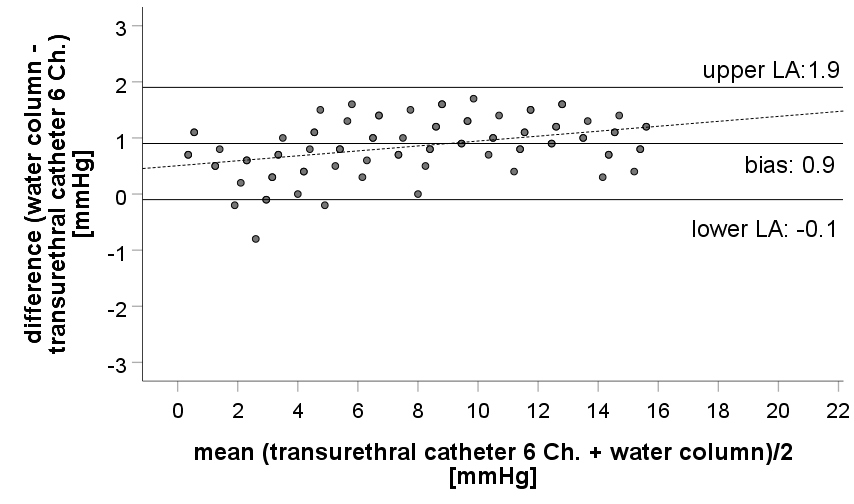** |
|  | **8 Ch.** | **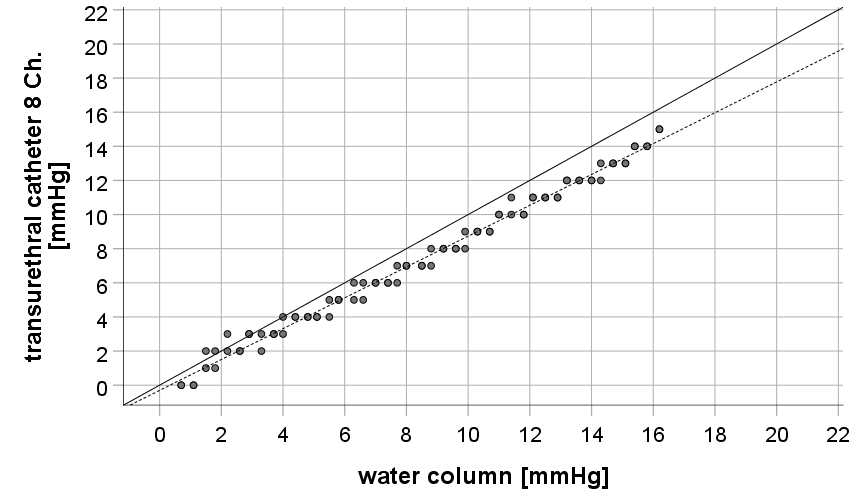** | **D** | **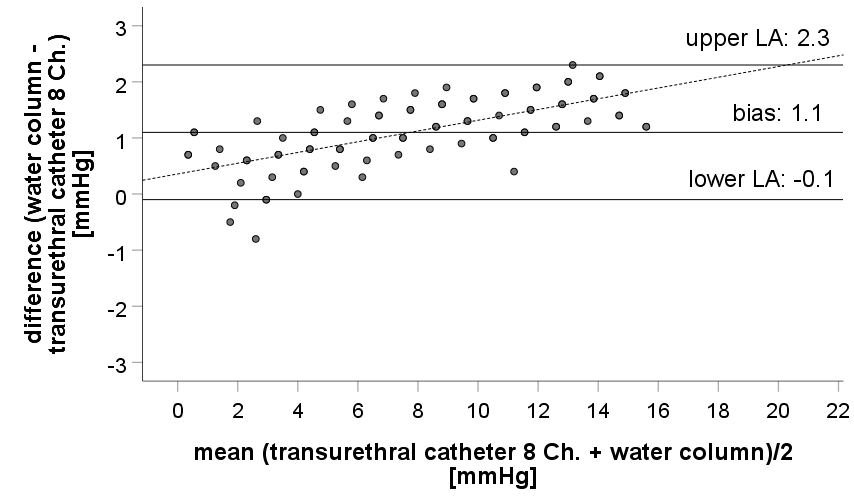** |

**(A+B)** Scatter plot and Bland-Altman plot of paired **in-vitro** IVP measurements via **gastric tubes sized 5 Ch. (A) and 8 Ch. (B)** compared to the water column of the container. (A1) Scatter plot (5 Ch. gastric tube vs. water column) with the solid line representing linear regression and the dashed line representing the line of identity. (A2) Bland–Altman plot: Mean bias±precision 0.8±0.3mmHg, LOA 0.2 to 1.4mmHg, PE 8%, MAPE±SD 6±13 %. (B1) Scatter plot (8 Ch. gastric tube vs. water column) with the solid line representing linear regression and the dashed line representing the line of identity. (B2) Bland–Altman plot: Mean bias±precision 0.1±0.4 mmHg; LOA -0.7 to 0.9mmHg, PE 10%, MAPE± 8±16%.

**(C+D)** Scatter plot and Bland-Altman plot of paired **in-vitro** IVP measurements via **transurethral catheters sized 6 + 8 Ch.** compared to the water column of the container. (C1) Scatter plot (6 Ch. transurethral catheter vs. water column) with the solid line representing linear regression and the dashed line representing the line of identity. (C2) Bland–Altman plot: Mean bias ±precision 0.9±0.5 mmHg, LOA -0.1 to 1.9 mmHg, PE 13%, MAPE±SD 9 ±13%. (D1) Scatter plot (8 Ch. transurethral catheter vs. water column) with the solid line representing linear regression and the dashed line representing the line of identity. (D2) Bland–Altman plot: Mean bias±precision 1.1±0.6 mmHg; LOA -0.1 to 2.3mmHg, PE 15%, MAPE±SD 14±22%.

# Additional File References:

1. De Waele JJ, Cheatham ML, Malbrain, M L N G et al (2009) Recommendations for research from the International Conference of Experts on Intra-abdominal Hypertension and Abdominal Compartment Syndrome. Acta Clin Belg 64:203-209. <https://doi.org/>10.1179/acb.2009.036

2. Lee S, Anderson J, Kraut E, Wisner D, Wolfe B (2002) A simplified approach to the diagnosis of elevated intra-abdominal pressure. J Trauma 52:1169-1172. <https://doi.org/>10.1097/00005373-200206000-00024

3. Schachtrupp A, Tons C, Fackeldey V, Hoer J, Reinges M, Schumpelick V (2003) Evaluation of two novel methods for the direct and continuous measurement of the intra-abdominal pressure in a porcine model. Intensive Care Med 29:1605-1608. <https://doi.org/>10.1007/s00134-003-1847-3

4. Wauters J, Spincemaille L, Dieudonne AS, Van Zwam K, Wilmer A, Malbrain ML (2012) A Novel Method (CiMON) for Continuous Intra-Abdominal Pressure Monitoring: Pilot Test in a Pig Model. Crit Care Res Pract 2012:181563. <https://doi.org/>10.1155/2012/181563

5. Correa Martín L, Castellanos G, García M, Sánchez Margallo FM (2013) Renal consequences of intraabdominal hypertension in a porcine model. Search for the choice indirect technique for intraabdominal pressure measurement. Actas Urol Esp 37:273-279. <https://doi.org/>10.1016/j.acuro.2012.06.001

6. Wise RD, Rodseth RN, Correa-Martin L, Sanchez Margallo FM, Becker P, Castellanos G, Malbrain M L G N (2017) Correlation between different methods of intraabdominal pressure monitoring in varying intraabdominal hypertension models. S Afr J Crit Care 33:15-18. <https://doi.org/>DOI:10.7196/SAJCC.2017.v33i1.327

7. Collee GG, Lomax DM, Ferguson C, Hanson GC (1993) Bedside measurement of intra-abdominal pressure (IAP) via an indwelling naso-gastric tube: clinical validation of the technique. Intensive Care Med 19:478-480. <https://doi.org/>10.1007/BF01711092

8. Turnbull D, Webber S, Hamnegard CH, Mills GH (2007) Intra-abdominal pressure measurement: validation of intragastric pressure as a measure of intra-abdominal pressure. Br J Anaesth 98:628-634. <https://doi.org/>98/5/628 [pii]

9. De Waele J, Berrevoet F, Reyntjens K, Pletinckx P, De Laet I, Hoste E (2007) Semicontinuous intra-abdominal pressure measurement using an intragastric Compliance catheter. Intensive Care Med 33:1297-1300. <https://doi.org/>10.1007/s00134-007-0682-3

10. Otto J, Kaemmer D, Biermann A, Jansen M, Dembinski R, Schumpelick V, Schachtrupp A (2008) Clinical evaluation of an air-capsule technique for the direct measurement of intra-abdominal pressure after elective abdominal surgery. BMC Surg 8:18. <https://doi.org/>10.1186/1471-2482-8-18

11. Davis P, Koottayi S, Taylor A, Butt W (2005) Comparison of indirect methods of measuring intra-abdominal pressure in children. Intensive Care Med 31:471-475. <https://doi.org/>10.1007/s00134-004-2539-3

12. De Potter, Tom J R, Dits H, Malbrain MLNG (2005) Intra- and interobserver variability during in vitro validation of two novel methods for intra-abdominal pressure monitoring. Intensive Care Med 31:747-751. <https://doi.org/>10.1007/s00134-005-2597-1

13. Malbrain ML, De laet I, Viaene D, Schoonheydt K, Dits H (2008) In vitro validation of a novel method for continuous intra-abdominal pressure monitoring. Intensive Care Med 34:740-745. <https://doi.org/>10.1007/s00134-007-0952-0

14. Garcia AF, Chica Yanten J, Sanchez AI, Aldana JL, Mejia JH, Burbano D, Salazar C (2020) Bench Validation of a Handcrafted Prototype Catheter for Intra-gastric Pressure Monitoring. World J Surg. <https://doi.org/>10.1007/s00268-020-05392-7

15. Kirkpatrick AW, Roberts DJ, De Waele J et al (2013) Intra-abdominal hypertension and the abdominal compartment syndrome: updated consensus definitions and clinical practice guidelines from the World Society of the Abdominal Compartment Syndrome. Intensive Care Med 39:1190-1206. <https://doi.org/>10.1007/s00134-013-2906-z

16. Bland JM,Altman DG (1986) Statistical methods for assessing agreement between two methods of clinical measurement. Lancet 1:307-310. <https://doi.org/>10.1016/S0140-6736(86)90837-8

17. de Myttenaere A, Golden B, Le Grand B, Rossi F (2016) Mean Absolute Percentage Error for regression models. Neurocomputing. <https://doi.org/><https://doi.org/10.1016/j.neucom.2015.12.114>
